# Increase of Severe Pulmonary Infections in Adults Caused by M1_UK_ *Streptococcus pyogenes*, Central Scotland, UK

**DOI:** 10.3201/eid2908.230569

**Published:** 2023-08

**Authors:** Peter J.B. Davies, Clark D. Russell, Anna-Rose Morgan, Surabhi K. Taori, Diane Lindsay, Roisin Ure, Derek Brown, Andrew Smith

**Affiliations:** Glasgow Royal Infirmary, Glasgow, Scotland, UK (P.J.B. Davies, D. Lindsay, R. Ure, D. Brown A. Smith);; University of Edinburgh, Edinburgh, Scotland, UK (C.D. Russell);; Royal Infirmary of Edinburgh, Edinburgh (C.D. Russell, S.B. Taori);; Queen Elizabeth University Hospital, Glasgow (A,-R. Morgan); University of Glasgow, Glasgow (A. Smith)

**Keywords:** streptococci, group A Streptococcus, necrotizing pneumonia, M1_UK_, Streptococcus pyogenes, Scotland, United Kingdom, bacteria, respiratory infections

## Abstract

We characterized the epidemiology, host–pathogen characteristics, and outcomes of severe adult pulmonary *Streptococcus pyogenes* infections that coincided with a high community caseload in central Scotland, UK. The pulmonary infections had high illness and death rates and were associated with socioeconomic deprivation, influenza A co-infection, and the M1_UK_ lineage of *S. pyogenes*.

The association between respiratory viruses and secondary invasive pulmonary bacterial disease is recognized, but the proportion of pulmonary invasive group A *Streptococcus* (PiGAS) infections after seasonal influenza is low compared with those for other bacterial pathogens (e.g., *Streptococcus pneumoniae*, *Haemophilus influenzae*, and *Staphylococcus aureus*) (*1*,*2*). However, PiGAS has been shown to complicate epidemics of measles and, notably, the 1918–1919 influenza pandemic (*3*).

Winter 2022–23 saw a marked increase in influenza and associated group A *Streptococcus* (GAS) infections in the United Kingdom as well as globally (https://www.gov.uk/government/publications/group-a-streptococcal-infections-activity-during-the-2022-to-2023-season/group-a-streptococcal-infections-second-update-on-seasonal-activity-in-england-2022-to-2023). In autumn 2022, an unusually high number of pediatric GAS pleural empyema cases associated with human metapneumovirus co-infection was described in Scotland (*4*). After similar cases were observed in adults, we aimed to characterize the burden of PiGAS in adults and contrast it to published and local historical data.

## The Study

We identified patients with pulmonary samples or blood cultures found positive for *S. pyogenes* by the National Health Service (NHS) of Greater Glasgow and Clyde (GGC), which serves a population of 1.4 million, during December 1, 2017–November 31, 2022, through the Laboratory Information Management System. We identified the same specimens from NHS Lothian (population 850,000) and GGC during December 1, 2022–February 28, 2023 (https://www.nrscotland.gov.uk/statistics-and-data/statistics/statistics-by-theme/population/population-estimates/mid-year-population-estimates/mid-2021).

Samples assessed were sputum (inpatient), pleural fluid, endotracheal aspirate, bronchoalveolar lavage pulmonary tissue (postmortem), and blood cultures. We also identified *S. pyogenes* by molecular techniques (i.e., specific GAS PCR testing and 16s PCR). We defined cases as definite or probable PiGAS. A definite case required microbiologic criteria (e.g., *S. pyogenes* identified in blood or deep respiratory or pleural sample) and radiologic criteria (e.g., multifocal consolidation or pleural effusion or empyema or parenchymal necrosis) to be present. Probable cases were those with sputum samples in the microbiologic criteria and unifocal consolidation in the radiologic criteria, capturing patients meeting both criteria but not those of a definite case.

We extracted demographic, clinical, and laboratory data from electronic patient records. We derived Scottish Index of Multiple Deprivation (SIMD) scores by using postcodes. We also calculated Charlson comorbidity index scores. We referred *S. pyogenes* isolates to the Scottish Microbiology Reference Laboratory for M-typing and Illumina short read sequencing (*5*). We used an annotated whole genome–core genome multilocus sequence typing approach to compare isolate sequence data with a publicly available core genome multilocus sequence typing scheme implemented in Ridom SeqSphere+ version 8.5.1 (http://www.ridom.de/seqsphere), enabling assignment to the M1 lineages described in the European Nucleotide Archive (*5*,*6*). Currently, >200 recorded M-types are used to identify outbreaks and determine cluster management; M1 is most commonly associated with invasive disease (*7*).

**Figure 1:**
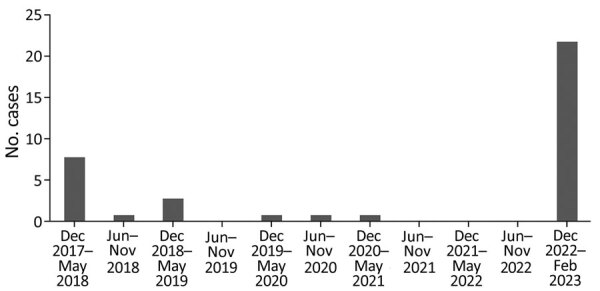
Monthly incidence of pulmonary invasive group A *Streptococcus* infections in adults >18 years of age, National Health Service Greater Glasgow and Clyde region, central Scotland, UK, December 2017–February 2023.

NHS GGC and Lothian provides public healthcare for ≈39% of Scotland’s population of 5.49 million (https://www.nrscotland.gov.uk/statistics-and-data/statistics/statistics-by-theme/population/population-estimates/mid-year-population-estimates/mid-2021). Like other countries, the United Kingdom is undergoing a resurgence of *S. pyogenes* infections that began in September 2022 (https://www.who.int/emergencies/disease-outbreak-news/item/2022-DON429). We identified 38 patients with PiGAS (30 definite, 8 probable) in the 3-month study period (22 in GGC, 16 in Lothian). In the previous 5-year period in GGC, we identified 15 cases (12 definite, 3 probable, 1 metastatic) (Figure 1).

We observed no significant difference in the median age between the 2022–23 cohort and historical records (45 [interquartile range (IQR) 26] years) vs. 57 [IQR 23.5] years). Both groups were healthy at baseline, having a median Charlson comorbidity index score of 0. We noted no significant difference in chronic respiratory underlying conditions. The 2022–23 cohort was associated with a more deprived SIMD postcode (median SIMD score of 3 vs. 5 for historical records). Other demographic, biochemical, and hematologic characteristics were comparable (Table). The PiGAS syndrome exhibits substantial leukopenia (median 0.53 [IQR 0.3–1] × 10^9^ cells/L) with a normal median white cell count (9.5 [IQR 4–18.7] × 10^9^ cells/L). Although respiratory symptoms predominated, diarrhea was reported in 18.4% (7/38) of cases in the 2022–23 outbreak. Microbiologically confirmed empyema was common, and a greater number of 2022–23 patients received an intercostal drain and exhibited radiologic evidence of empyema or pleural effusion (Table). Those characteristics, along with multifocal consolidation, were the radiologic hallmarks of PiGAS; cavitation also was common. A lower proportion of patients in the historical cohort had chest computed tomography results, precluding greater sensitivity (47% vs. 76%).

**Table:** Characteristics associated with a historical cohort (2017–2022) and a recent epidemic of severe pulmonary infections in adults (2022–23) caused by M1_UK_ *Streptococcus pyogenes*, central Scotland, UK*

Characteristic	2017–2022, GGC only	2022–2023, GGC and Lothian
Demographic		
Total no. PiGAS cases	15	38
Median age, y (IQR)	57 (42.5–66)	45 (37–63)
Sex, no (%)		
M	13 (87)	20 (53)
F	2 (6)	18 (47)
Median SIMD score (IQR)	5 (3.5)	3 (1–5)
Blood parameters at admission		
Median C-reactive protein, mg/L (IQR)†	293 (198–360)	328 (172–410)
Median leukocyte count, × 10^9^ cells/L (IQR)‡	9.5 (3.1–13.7)	9.45 (4.1–22.1)
Median lymphocyte count, × 10^9^ cells/L (IQR)§	0.5 (0.3–0.7)	0.54 (0.31–1.35)
Radiographic, no. (%)		
Pleural effusion	8 (53)	23 (61)
Focal consolidation	3 (20)	9 (24)
Multifocal consolidation	11 (73)	28 (74)
Cavitation or necrosis	2 (13)	7 (18)
Background		
Chronic respiratory disease, no (%)	7 (47)	9 (24)
Smoker, no (%)	2 (13)	10 (26)
No past medical history, no (%)	4 (26.7)	17 (44.7)
Median CCI score (IQR)	0 (0–3)	0 (0–3)
Viral co-infection		
No. tested	12	33
Influenza A	1	19
Influenza B	3	0
Parainfluenza 1	1	0
Metapneumovirus	2	4
RSV	0	1
Adenovirus	0	1
None detected	5	8
Outcomes		
ICU admission, no (%)	8 (53)	21 (55)
Median ICU length of stay, d (IQR)	5 (2–9.8)	15 (3.5–27)
Vasopressors, no. (%)	4 (26)	15 (39)
Invasive mechanical ventilation, no. (%)	7 (46)	16 (42)
Death, no (%)	3 (20)	6 (16)
Median days from admission to death (IQR)	1 (1–2)	1 (0–2)
M type		
No. typed	8	25
1.0	3	24
12.0	1	1
3.93	1	0
44.0	1	0
5.23	2	0

*Historical cohort comprises cases identified among the population served by NHS Greater Glasgow and Clyde; cases from the recent epidemic are those identified among populations served by NHS Greater Glasgow and Clyde and NHS Lothian. CCI, Charlson comorbidity index; ICU, intensive care unit; IQR, interquartile range; NHS, National Health Service; PiGAS, pulmonary invasive group A *Streptococcus*; RSV, respiratory syncytial virus; SIMD, Scottish Index of Multiple Deprivation. †Reference range <5 mg/L. ‡Reference range 4–10 × 10^9^ cells/L. §Reference range 1.1–5.0 × 10^9^ cells/L.

**Figure 2:**
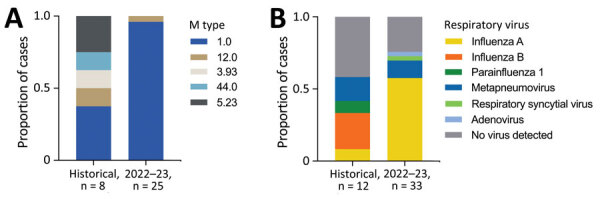
Microbiologic features of pulmonary invasive group A *Streptococcus* infections, central Scotland, UK, December 2017–February 2023. A) *Streptococcus pyogenes* isolate M type results, where available, comparing historical data (December 2017–November 2022) with 2022–23 cohort (December 2022–February 2023). B) Results of respiratory virus testing, where available, comparing historical data with 2022–23 cohort.

During 2017–2022, co-infecting respiratory viruses were varied, but in 2022–23, most patients tested positive for influenza A (Table; Figure 2). Only half of patients underwent an extended respiratory viral screen. Of typed *S. pyogenes* isolates from the current outbreak, 24/25 belonged to the M1_UK_ lineage, contrasting with the historical cohort that involved a mixture of M types (M1, M12, M3.93, M44, and M5.23).

## Conclusions

Europe is experiencing an increased incidence of invasive GAS disease (*4*). We report an unusually high incidence of severe PiGAS in adults from central Scotland. We also note an additional strong association with influenza A co-infection and the near-complete dominance of M1_UK_, contrasting with local and published precedent. M1 comprised 38% of adult and 58% of pediatric invasive GAS referrals in England during 2022–23, in contrast to the 96% we report (https://www.gov.uk/government/publications/group-a-streptococcal-infections-activity-during-the-2022-to-2023-season/group-a-streptococcal-infections-second-update-on-seasonal-activity-in-england-2022-to-2023). The clinical phenotype of severe, often rapidly fatal PiGAS disease in young healthy adults parallels outbreaks described around World War I and in institutional facilities (*3*,*8*). The M1_UK_ lineage is emerging as a dominant lineage within M1 worldwide and often associated with invasive GAS (*9*–*12*).

The pathophysiology of PiGAS after a respiratory viral infection (influenza A in our cohort) is incompletely understood. In vitro and in vivo studies suggest prior influenza A infection increases both GAS adherence and internalization by binding to viral hyaluronic acid on the infected host cell surface, which is followed by increases in the abundance of, and access to, bacterial receptors and the GAS ligands fibrinogen and fibronectin (*13*). Influenza B is also implicated, both in the literature and locally during 2017–2018 (*14*).

M1 has an established association with severe disease (*7*). In particular, the M1_UK_ strain appears to have an enhanced capability for transmission and virulence and is now the predominant strain in the United Kingdom (*6*). This strain exhibits a hypervirulent phenotype because of greater expression of streptococcal pyrogenic exotoxin A than global M1 strains. Case reports of severe rapidly fatal M1 PiGAS in young healthy patients echo outcomes seen in our cohort (*15*).

Modern molecular techniques have revolutionized our ability to investigate patterns of disease (e.g., widespread availability of rapid point-of-care tests for COVID-19 and influenza). We are experiencing a major outbreak of *S. pyogenes* infections with an unusual predilection for severe pulmonary disease in addition to the usual manifestations of disease by this pathogen, including distinctive viral and M-type associations in the winter and spring of 2022–23. The PiGAS phenotype we describe is similar to those from more sporadic reports identified from a review of published case series of severe pulmonary infections from *S. pyogenes* (Appendix). Historical outbreaks probably underreported coexistent viral infections because of a lack of accessible point-of-care tests. Similarly, only half of patients had an extended viral respiratory screen, and we therefore risk underreporting metapneumovirus cases, an agent notable locally in GAS empyema in children immediately before December 2022 (*4*). Although our cohort is small, it is comparatively large compared with the few described in the literature and notable for the short timeframe of cases captured.

Our study highlights a new aggressive pattern of *S. pyogenes* infections linked to the dominant circulating M1_UK_ strain, manifesting as severe pulmonary disease and having a strong association with influenza A co-infection. Clinicians and public health officials need to be vigilant of such clinical manifestations while rates of iGAS remain high.

Appendix Additional information about increase of severe pulmonary infections in adults caused by M1_UK_ *Streptococcus pyogenes*, Central Scotland.
