# Supplementary material for: Increase of Severe Pulmonary Infections in Adults Caused by M1UK Streptococcus pyogenes, Central Scotland, UK
Source: Emerg Infect Dis. 2023 Aug;29(8):1638–42. doi: 10.3201/eid2908.230569 (PMC10370863; doi:10.3201/eid2908.230569)
Supplement: Appendix — Additional information about increase of severe pulmonary infections in adults caused by M1UK Streptococcus pyogenes, Central Scotland. [file 23-0569-Techapp-s1.pdf]

Article DOI: https://doi.org/10.3201/eid2908.230569

EID cannot ensure accessibility for supplementary materials supplied by authors. Readers who have difficulty accessing supplementary content should contact the authors for assistance.

Increase of Severe Pulmonary Infections in Adults Caused by M1UK Streptococcus pyogenes, Central Scotland, UK

Appendix

Appendix Table. Tabulates demographic, clinical and laboratory details of established case series of PiGAS*

Study	PiGAS no.	Time period	Median age, y	Male sex, %	No PMH, no. (%)	Empyema, no. (%)	Cavitation, no. (%)	Predominant viral co-infection, no. (%)	M-type 1.0, no. (%)	Mortality, no. (%)	Comment
Switzerland (*1*)	2	2007–2008	38	0	0	50	0	Influenza B, 2 (100)	NA	2 (100)	Case reports
Italy (*2*)	3	2023	65	66	100	100	NA	Nil	3 (100)	2 (100)	Case reports
Yorkshire, UK (*3*)	17	1981–1997	52	47	47	17.6	5.9	Influenza A,1 (5.9)	9 (53)	8 (47)	6 had viral prodrome
San Diego, USA (*4*)	34	2002	19	NA	0 (100)	5 (14.7)	0	Adenovirus (5, unclear if all PiGAS)	0 (all M3)	0 (0)	Military facility
Gipuzkoa, Spain (*5*)	40	2006−2015	58.4	63.5	32.5	27.5	10	Influenza A, 3; influenza B, 1	17 (46.3)	8 (20)	
Ontario, Canada (*6*)	222	1992–2000	56	57.7	39	19	1.3	Influenza A, 5 (0.2)	74 (38)	85 (38)	Nursing home outbreaks

*Includes some partial large volume retrospective data that distinguished little between iGAS and PiGAS. PMH, past medical history.

References

1. Aebi T, Weisser M, Bucher E, Hirsch HH, Marsch S, Siegemund M. Co-infection of Influenza B and Streptococci causing severe pneumonia and septic shock in healthy women. BMC Infect Dis. 2010;10:308. PubMed https://doi.org/10.1186/1471-2334-10-308

2. Santagati M, Spanu T, Scillato M, Santangelo R, Cavallaro F, Arena V, et al. Rapidly fatal hemorrhagic pneumonia and group A *Streptococcus* serotype M1. Emerg Infect Dis. 2014;20:98–101. PubMed https://doi.org/10.3201/eid2001.130233

3. Barnham M, Weightman N, Anderson A, Pagan F, Chapman S. Review of 17 cases of pneumonia caused by *Streptococcus pyogenes*. Eur J Clin Microbiol Infect Dis. 1999;18:506–9. PubMed https://doi.org/10.1007/s100960050333

4. Crum NF, Russell KL, Kaplan EL, Wallace MR, Wu J, Ashtari P, et al. Pneumonia outbreak associated with group a *Streptococcus* species at a military training facility. Clin Infect Dis. 2005;40:511–8. PubMed https://doi.org/10.1086/427502

5. Tamayo E, Montes M, Vicente D, Pérez-Trallero E. *Streptococcus pyogenes* pneumonia in adults: clinical presentation and molecular characterization of isolates 2006–2015. PLoS One. 2016;11:e0152640. PubMed https://doi.org/10.1371/journal.pone.0152640

6. Muller MP, Low DE, Green KA, Simor AE, Loeb M, Gregson D, et al.; Ontario Group A Streptococcal Study. Clinical and epidemiologic features of group a streptococcal pneumonia in Ontario, Canada. Arch Intern Med. 2003;163:467–72. PubMed https://doi.org/10.1001/archinte.163.4.467
